# Metabotypes of response to bariatric surgery independent of the magnitude of weight loss

**DOI:** 10.1371/journal.pone.0198214

**Published:** 2018-06-01

**Authors:** Magali Palau-Rodriguez, Sara Tulipani, Anna Marco-Ramell, Antonio Miñarro, Olga Jáuregui, Alex Sanchez-Pla, Bruno Ramos-Molina, Francisco J. Tinahones, Cristina Andres-Lacueva

**Affiliations:** 1 Biomarkers & Nutrimetabolomic Laboratory, Nutrition, Department of Food Science and Gastronomy, XaRTA, INSA-UB, Campus Torribera, Faculty of Pharmacy and Food Science, University of Barcelona, Barcelona, Spain; 2 CIBER Fragilidad y Envejecimiento Saludable [CIBERfes], Instituto de Salud Carlos III [ISCIII], Madrid, Spain; 3 Biomedical Research Institute [IBIMA], Service of Endocrinology and Nutrition, Malaga Hospital Complex [Virgen de la Victoria], Málaga, Spain; 4 Department of Genetics, Microbiology and Statistics, Faculty of Biology, University of Barcelona, Barcelona, Spain; 5 Scientific and Technological Centers of the University of Barcelona (CCIT-UB), Barcelona, Spain; 6 Statistics and Bioinformatics Unit, Vall d’Hebron Institut de Recerca [VHIR], Barcelona, Spain; 7 CIBER Fisiopatología de la Obesidad y Nutrición [CIBERobn], Instituto de Salud Carlos III [ISCIII], Barcelona, Spain; Instituto de Investigacion Sanitaria INCLIVA, SPAIN

## Abstract

**Objective:**

Bariatric surgery is considered the most efficient treatment for morbid obesity and its related diseases. However, its role as a metabolic modifier is not well understood. We aimed to determine biosignatures of response to bariatric surgery and elucidate short-term metabolic adaptations.

**Methods:**

We used a LC- and FIA-ESI-MS/MS approach to quantify acylcarnitines, (lyso)phosphatidylcholines, sphingomyelins, amino acids, biogenic amines and hexoses in serum samples of subjects with morbid obesity (n = 39) before and 1, 3 and 6 months after bariatric surgery. K-means cluster analysis allowed to distinguish metabotypes of response to bariatric surgery.

**Results:**

For the first time, global metabolic changes following bariatric surgery independent of the baseline health status of the subjects have been revealed. We identify two metabolic phenotypes (metabotypes) at the interval 6 months-baseline after surgery, which presented differences in the levels of compounds of urea metabolism, gluconeogenic precursors and (lyso)phospholipid particles. Clinically, metabotypes were different in terms of the degree of improvement in insulin resistance, cholesterol, low-density lipoproteins and uric acid independent of the magnitude of weight loss.

**Conclusions:**

This study opens new perspectives and new hypotheses on the metabolic benefits of bariatric surgery and understanding of the biology of obesity and its associated diseases.

## Introduction

Over the last few decades bariatric surgery has been used as a powerful “disease modifier” for the treatment of morbid obesity, not merely as a strategy for weight loss [1]. To date, bariatric surgery is the most successful treatment for weight loss, metabolic control and effective prevention, remission or delay of type 2 diabetes progression [2].

The metabolic co-morbidities of obesity such as diabetes improve after bariatric surgery even before weight loss occurs. However, why this happens is still unclear. Previous investigations focused on metabolic patterns in a specific view such as according to the type of surgery received [3] or the remission state of the subject before the intervention [4]. Few studies have been carried out to explore signatures after bariatric surgery and their association with the heterogeneity of metabolic improvement [5]. Therefore, unveiling different metabolic fingerprints following bariatric surgery in the earliest stages might help to elucidate the physiological pathways of insulin resistence and the onset of the related co-morbidities.

Metabolomics has emerged in biomedical investigation as a tool to uncover obesity-associated metabolic diseases and could give insights into the prognosis of weight loss intervention through the study of the metabolome. The study of the metabolome through high-throughput mass spectrometry provide a comprehensive overview of the metabolic processes in a given moment and define metabolic phenotypes (metabotypes) of responses to a treatment or an intervention [6].

Hence, the aims of the present study are to: 1) discriminate metabolic signatures of response to bariatric surgery associated with the baseline condition of the patient, “metabolically unhealthy” or “metabolically healthy”; 2) elucidate metabotypes of response to bariatric surgery according to their metabolic evolution; and 3) associate these changes with different degrees of metabolic improvement according to anthropometric and clinical parameters. The baseline condition of the patients dissipate after the surgery. We have defined two metabotypes of response independently of the gender, age or the amount of weight loss but dependent of insulin resistance, cholesterol and uric acid levels. Despite further studies are needed, our results open new hypothesis in the study of obesity and provide a comphensive view of the metabolic changes after the surgery.

## Materials and methods

Serum samples from 39 patients with morbid obesity (body mass index (BMI) > 40 kg/m^2^) were collected before and 1 (T1), 3 (T3) and 6 (T6) months after bariatric surgery at the Virgen de la Victoria University Hospital (Malaga, Spain). The availability of the samples after the surgery were 38 (T1), 34 (T3), 27 (T6) respectively. Twenty-six patients underwent a Roux-en-Y gastric bypass (RYGB) procedure and 13 patients sleeve gastrectomy (SG). All the patients were adults (19–59 years old), comprising 27 females and 12 males with > 10-year history of obesity. The inclusion and exclusion criteria are detailed in [7]. Briefly, the exclusion criteria were the intake of antidiabetic, corticosteroid or antibiotic drug treatment; the presence of acute or chronic infection, a history of alcohol abuse or drug dependence, a history of cancer. Other treatment including anti-inflammatory, antihypertensive and anti-cholesterolemic agents were recorded.

Initially, subjects were stratified according to their degree of metabolic syndrome, as defined by the Adult Treatment Panel III criteria [8]: metabolically healthy (MH) subjects with ≤ 2 criteria (n = 21) and metabolically unhealthy (MU) subjects with ≥ 3 criteria (n = 18).

### Anthropometric and clinical parameters

Anthropometric and clinical parameters were measured using standardized techniques [9,10]. In this work we report: a) anthropometric markers: body weight, BMI, waist circumference, hip circumference and waist-hip ratio; b) markers of glucose regulation: glycated haemoglobin A1c (HbA1c); plasma fasting glucose and insulin, Homeostatic Model Assessment (HOMA-IR score = fasting insulin x fasting glucose/22.5 AU; c) blood pressure markers: diastolic and systolic blood pressure; d) blood lipid markers: total cholesterol (CHOL), very low-density lipoprotein (VLDL), low-density lipoprotein (LDL) and high-density lipoprotein (HDL), and triglycerides (TG); e) hormones: leptin and adiponectin; f) kidney impairment/disease markers: urea, uric acid and creatinine; g) other standard biochemical markers including liver enzymes, C-reactive protein (CRP).

The protocol was approved by the local Ethics and Research Committee (Hospital Universitario Virgen de la Victoria, Málaga) and all participants provided written informed consent.

### Metabolomic analysis

Amino acids and biogenic amines were acquired by liquid chromatography (10 μl injection volume) and acylcarnitines, glycerophospholipids, sphingolipids and total hexoses by flow injection analysis (20 μl injection volume), both coupled with tandem mass spectrometry in positive and negative electrospray ionization modes (S1 Table). All the analyses were performed on a QTRAP 6500 System (AB Sciex, Framingham, MA).

To avoid run-order effects, serum samples were analysed in a randomized batch format. Quality controls were analysed throughout the whole run to control the stability and performance of the system and evaluate the quality of the acquired data [11]. Metabolites were quantified by multiple reaction monitoring, by reference to multipoint calibration curves and/or in combination with the use of stable isotope-labelled and other internal standards, to compensate for matrix effects [12].

Isotopic correction was performed with the software Met*IDQ*^™^ (Biocrates Life Sciences AG). The limits of detection (LOD) and lower (LLOQ) and upper limits of quantification were experimentally determined.

### Data pre-processing

Metabolite measures below the LOD or LLOQ in more than 25% of subjects at any time point and/or with high analytical variances (CV>25% in the quality control) were excluded from further analyses (S1 Table).

Values below the LOD and LLOQ were imputed using theoretical LOD and LLOQ values (LOD/√2 and LLOQ/√2) within every metabolite level across all the samples. Data were log-transformed. The missing values were dealt with using the K-means nearest neighbour imputation method (K = 10). Finally, data were subjected to auto-scaling.

### Statistical analysis

T-test for independent samples was used to compare anthropometric/clinical and metabolic variables between patient groups in the preoperative state (baseline). Fisher’s exact tests were performed to explore the distribution of males and females. Multiple factor analysis (MFA) was used to explore how the metabolic variables evolved over time, taking into account the three following temporal increments: T1–T0, T3–T1 and T6–T3. Further information about MFA is available as Supporting Information and in [13]. Principal component analysis (PCA) was performed to provide single snapshots of each increment of time in respect to the baseline (T6–T0, T3–T0 and T1–T0). Global metabolic post-surgery changes were evaluated by MFA through the projection of each subject on the individual factor map and each metabolite in the correlation circle, represented as vectors.

Linear mixed models were performed to analyse: 1) the track of single metabolites and anthropometric/clinical variables after the intervention; 2) the changes of these variables at each pre- and post-surgery time point; 3) the influence of the type of surgery; and 4) the baseline health status throughout the evolution of the patient after bariatric surgery.

In order to associate the different patterns of response observed on the MFA’s individual factor map, metabolic clusters were determined at T6–T0 with K-means cluster analysis (Supporting Information). The most responsible variables for cluster separation were the most correlated with the PCA (correlations r >0.75 or <−0.75 with the PC1). The application of sparse partial least squares discriminant analysis (sPLS-DA) confirmed them. The sparsity (*eta*) and number of hiding components (*K*) were chosen using a 10-fold cross-validation procedure and the predictability of the model was calculated on a leave-one-out basis. Linear mixed models also confirmed the discriminant metabolites and anthropometric/clinical differences when the *P* value of the interaction *time* x *cluster* was statistically different (p<0.05).

Pearson correlation coefficients were calculated to estimate the linear correlation among the discriminant metabolic variables and anthropometric/clinical variables in each metabolic cluster. Metabolite-clinical correlations were represented as a network and metabolite-metabolite correlations as a heatmap.

Enrichment analysis was performed using the bioinformatics tool ChemRich (Chemical Similarity Enrichment Analysis for Metabolomics) for each group of the study and with all the metabolites identified. The ChemRich statistical approach compares chemical similarities using the Medial Subject Headings database and Tanimoto chemical similarity coefficients to cluster metabolites into non-overlapping chemical groups. *P* values are obtained by employing the Kolmogorov-Smirnov test using the created clusters [14].

Since a potential effect on metabolite profiles has been described [15,16]: g*ender*, *age*, *drug intake and type of surgery* were used as covariables in the univariate analysis, and only *gender* and *age* in the un-supervised multivariate analysis (K-means and sPLS-DA analysis) to minimize the influence in the formation of the clusters.

All the *P* values reported in this study were adjusted by false discovery rate multiple testing (5%), based on the Benjamin-Hochberg procedure [17]. When linear mixed models were used, the evaluation of the models was performed using the Akaike information criterion [18]. All the statistical analyses and graphics were computed in R3.3.1 except the correlation network, which was performed using Cytoscape 3.3.0.

## Results

As shown in S1 Table we quantified 94 lipid metabolites (lysophosphatidylcholines (lysoPCs), acyl-alkyl phosphatidylcholines (PC ae), diacyl phosphatidylcholines (PC aa), sphingomyelins (SMs)) and 46 polar metabolites (acylcarnitines, amino acids and biogenic amines).

### General metabolomic patterns over time

**Fig 1:**
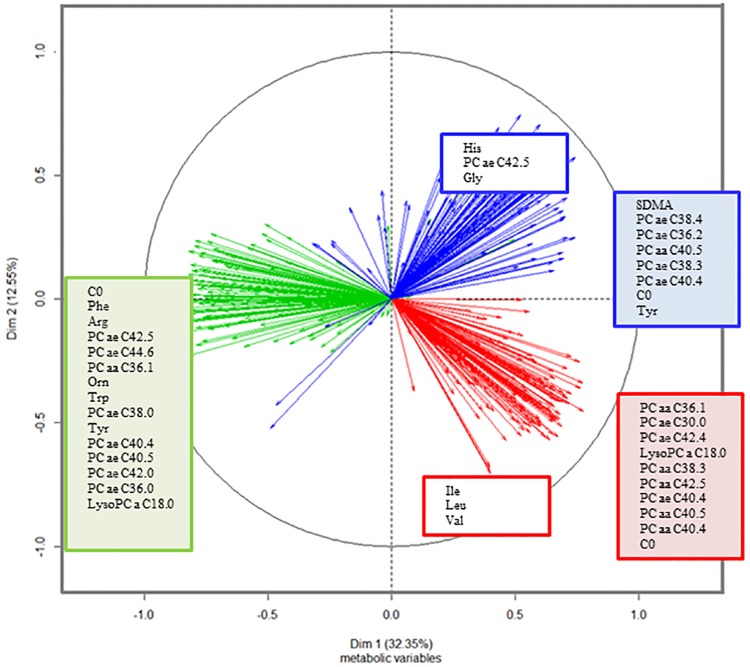
Correlation circle of metabolites in each increment of time in the first and second principal components: In red, metabolites correlated with T1–T0, in green, metabolites correlated with T3–T1, and in blue, metabolites correlated with T6–T3. Up to the most correlated 15 metabolites (correlation higher than 0.7 or smaller than -0.7) to each PC are shown. Metabolites correlated with the first dimension are written in the filled squares and those metabolites correlated with the second dimension are written in the white squares. Variables were adjusted by age and gender. The profiles of PC aa and PC ae and carnitine were also linked to T6–T3 in PC1. Carnitine negatively correlated with PC1 in the increment T3–T1 (r = -0.88, p<0.001). Changes in lysoPC a C18:0 inversely correlated with T1–T0 in PC1 (r = 0.77, p<0.001) and changes in T3–T1 in PC1 (r = -0.82, p<0.05). The increment T3–T1 correlated with a large profile of amino acids and certain PC aa and PC ae in PC1 (r<-0.85, p<0.05). Changes in tyrosine and PC ae C42:5 were kept in T6–T3 (r = 0.71 with PC2, p<0.01). Histidine and glycine correlated with the last time period (r>0.70 with PC2, p<0.001). Hence, T1–T0 was explained by changes in branched-chain amino acids (BCAAs, isoleucine, leucine and valine) (r<-0.7 with PC2, p<0.001) and PC ae and PC aa (r>0.7 with PC1, p<0.001).

We analysed all the quantified metabolites to distinguish a metabolic temporal pattern through MFA analysis. The two first principal components explained the 44.9% of the variability (S1A Fig). The MFA score plot separated the time intervals T3–T1 from T6–T3 and T1–T0 in the PC1 and PC2, but only the PC2 allowed the separation of T6–T3 from T1–T0 (S1B and S1C Fig). Fig 1 shows the most correlated metabolites for each increment of time and principal component (r>│0.7│).

### Temporal trends according to baseline status: Metabolically healthy (MH) versus metabolically unhealthy (MU) subjects

The individual factor map revealed different metabolic temporal profiles, but they were independent of the baseline health status of the subjects, defined by the Adult Treatment Panel III.

S2 Table shows the characteristics of each group at baseline. Clearly, at the baseline MU subjects presented higher levels of fasting glucose, HOMA-IR, VLDL and TG than MH. Metabolically, the MU group also had higher levels of alanine, glutamate, acylcarnitines C10:0 and C10:1, lysoPC a C16:0, C16:1 and C24:0 (S3 Table) than MH. However, the two groups presented a similar response to the bariatric surgery since no anthropometric/clinical or metabolic variables differed between groups (p>0.05 for *time* x *health*) (S4 and S5 Tables). A PCA corroborated these results (S2 Fig).

### Temporal trends according to K-means cluster analysis

#### Identification of temporal metabolomic metabotypes

**Fig 2:**
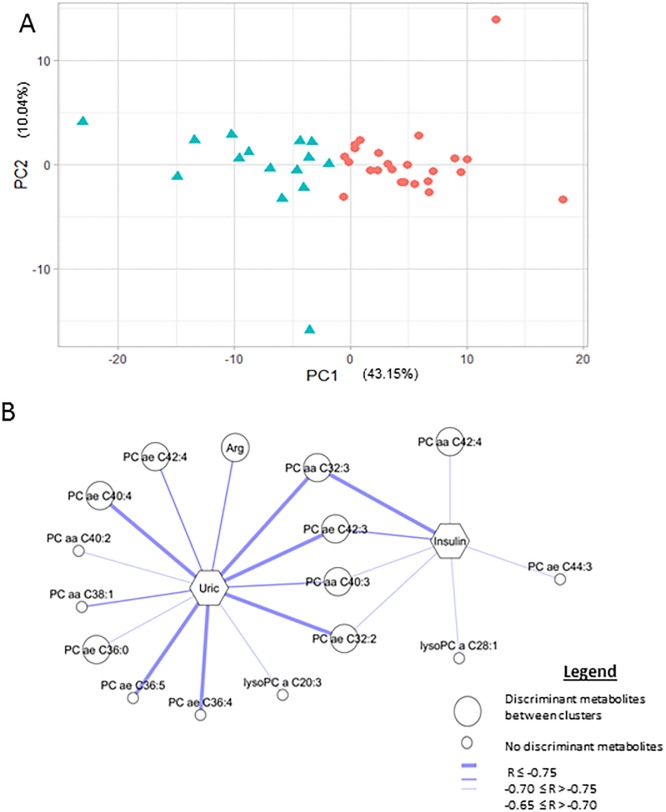
A. Scatter plot of cluster 1 (red dots) and cluster 2 (blue triangles) derived from K-means cluster analysis in the first (PC1) and second (PC2) principal components. B. Network of the correlations at T6–T0 of phenotype 1 between changes in uric acid (Uric) and fasting insulin (Insulin) and metabolites is represented. Only correlations with statistical significance and corrected by false-discovery rate are drawn. The most discriminant metabolites between clusters (correlation with PCA r > 0.75 or r < -0.75) are represented by a big circle, and other metabolites by a small circle. Correlations r ≤-0.75 are represented by a thick line, correlations -0.70 ≤ r > -0.75 are represented by a medium line and -0.65 ≤ r > -0.70 by a thin line. Arg: arginine, PC aa: diacyl phosphatidylcholines, PC ae: acyl-alkyl phosphatidylcholines, and lysoPC: lysophosphatidylcholines.

We performed a K-means cluster analysis with metabolite measurements in the time period T6–T0. The optimal number of clusters chosen according to the Calinski-Harabasz index (Supporting information) was two (cluster 1 and cluster 2). PC1 was the main contributor to separating the two clusters (43.2% of the total variability) (Fig 2A). Fifty-one metabolites correlated with PC1 (r>0.75 or <−0.75) (Table 1). Linear mixed models and a sPLS-DA, with an *eta* = 0.7, *K* = 1 and goodness of classification of 97.4%, confirmed these results.

**Table 1:** The most discriminating metabolites for K-means at 6-month increment versus baseline (sorted by family and alphabetical order).

Metabolites^a^	Cluster 1^b^	Cluster 2^b^	PC1 correlation^c^	*P*- Time^d^	*P*- Time X Cluster^d^	*P*- 6m X Cluster^d^
Alanine	0.45	-0.72	0.77	<0.05	<0.05	<0.05
Arginine	0.44	-0.71	0.77	<0.001	<0.05	<0.05
Histidine	0.38	-0.61	0.77	<0.001	<0.05	<0.05
Tyrosine	0.50	-0.81	0.78	<0.001	<0.05	<0.001
SDMA	0.51	-0.82	0.81	0.47	<0.05	<0.05
Carnitine	0.50	-0.80	0.81	<0.001	<0.05	<0.001
LysoPC a C16:0	0.46	-0.74	0.80	0.26	<0.001	<0.001
LysoPC a C18:0	0.51	-0.82	0.81	<0.001	<0.001	<0.001
LysoPC a C18:1	0.47	-0.76	0.79	<0.05	<0.05	<0.001
PC aa C28:1	0.55	-0.89	0.85	<0.001	<0.001	<0.001
PC aa C32:0	0.43	-0.69	0.81	0.24	<0.001	<0.001
PC aa C32:3	0.48	-0.76	0.81	0.09	<0.05	<0.001
PC aa C34:1	0.44	-0.71	0.82	0.82	<0.001	<0.001
PC aa C34:2	0.49	-0.78	0.88	<0.05	<0.001	<0.001
PC aa C36:0	0.55	-0.88	0.81	<0.001	<0.001	<0.001
PC aa C36:1	0.51	-0.81	0.82	<0.001	<0.001	<0.001
PC aa C36:2	0.48	-0.77	0.83	<0.001	<0.001	<0.001
PC aa C36:3	0.52	-0.82	0.82	<0.001	<0.001	<0.001
PC aa C36:4	0.38	-0.61	0.80	0.19	<0.05	<0.001
PC aa C38:0	0.58	-0.94	0.83	<0.05	<0.001	<0.001
PC aa C38:4	0.41	-0.65	0.79	<0.05	<0.001	<0.001
PC aa C38:5	0.40	-0.65	0.80	0.20	<0.05	<0.001
PC aa C38:6	0.51	-0.82	0.78	<0.05	<0.001	<0.001
PC aa C40:3	0.50	-0.81	0.79	<0.05	<0.001	<0.001
PC aa C40:4	0.55	-0.89	0.88	<0.001	<0.001	<0.001
PC aa C42:0	0.52	-0.84	0.75	0.48	<0.05	<0.001
PC aa C42:4	0.47	-0.75	0.76	<0.05	<0.001	<0.001
PC aa C42:5	0.47	-0.76	0.82	<0.05	<0.001	<0.001
PC ae C32:2	0.51	-0.81	0.89	<0.05	<0.05	<0.001
PC ae C34:0	0.56	-0.90	0.79	<0.05	<0.001	<0.001
PC ae C34:1	0.52	-0.83	0.90	<0.05	<0.001	<0.001
PC ae C36:0	0.55	-0.88	0.90	0.20	<0.001	<0.001
PC ae C36:1	0.53	-0.85	0.81	<0.001	<0.001	<0.001
PC ae C36:2	0.52	-0.84	0.81	<0.001	<0.001	<0.001
PC ae C38:0	0.52	-0.83	0.86	<0.001	<0.05	<0.001
PC ae C38:6	0.46	-0.74	0.77	<0.001	<0.05	<0.001
PC ae C40:4	0.55	-0.88	0.92	<0.001	<0.001	<0.001
PC ae C40:5	0.50	-0.80	0.86	0.74	<0.05	<0.001
PC ae C40:6	0.53	-0.85	0.85	0.34	<0.05	<0.001
PC ae C42:3	0.53	-0.85	0.83	<0.001	<0.001	<0.001
PC ae C42:4	0.49	-0.78	0.88	<0.001	<0.001	<0.001
PC ae C42:5	0.52	-0.84	0.89	0.31	<0.05	<0.001
PC ae C44:4	0.45	-0.71	0.78	<0.001	<0.05	<0.001
PC ae C44:5	0.47	-0.75	0.84	0.19	<0.05	<0.001
PC ae C44:6	0.49	-0.78	0.79	<0.05	<0.05	<0.001
SM (OH) C14:1	0.51	-0.81	0.87	0.23	<0.001	<0.001
SM (OH) C16:1	0.47	-0.75	0.78	0.28	<0.05	<0.001
SM C16:0	0.49	-0.79	0.87	0.52	<0.001	<0.001
SM C16:1	0.41	-0.66	0.80	0.30	<0.05	<0.001
SM C24:0	0.54	-0.86	0.76	<0.001	<0.001	<0.001
SM C24:1	0.46	-0.74	0.82	0.45	<0.05	<0.001

^a^ Metabolites with R>0.75 or R<-0.75 with the first component.

^b^Cluster 1 (Cl-1) and 2 (Cl-2) were derived from K-means cluster analysis of 140 different biomarkers. Values are changes 6 months respect baseline of variables after being adjusted by age and gender.

^c^ Correlation coefficient with the first principal component (43.15%). sPLSDA confirmed the results except His, PC.aa.C36.4, PC.aa.C38.4, PC.aa.C38.5.

^d^ *P* value derived from linear mixed model after log-transformed variables: TIME, effect of time over time (baseline, 1, 3 and 6 months after surgery), TIMExCLUSTER, interaction of both variables and 6mXCLUSTER, interaction of group at 6 months vs baseline. The model was adjusted by age, gender, drug intake and type of surgery and corrected for multiple testing by false discovery rate. Total n 39 patients, separate in Cluster 1 (Cl-1, n = 24) and Cluster 2 (Cl-2, n = 15) according K-means clustering at increment 6 month vs baseline. Numbers of serum samples 1, 3 and 6 months after surgery were as follows: Cl-1: 23, 21, 16 and Cl-2 15, 13, 11.

Abbreviations: LysoPC, lysophosphatidylcholine; PC aa, diacyl phosphatidylcholine; PC ae, acyl-alkyl phosphatidylcholine; SM, sphingomyelin; SMDA, symmetric dimethylarginine

In general, cluster 2 presented lower levels in all the selected metabolites at 6 months than at baseline, whereas cluster 1 had stable or even slightly higher levels than at baseline (S6 Table). In addition, the temporal pattern of cluster 1 was significantly different from cluster 2 for these metabolites. While the metabolite profiles of cluster 2 had a similar trend, specifically a sudden decrease followed by a slight recovery that quickly faded away, cluster 1 presented smoother metabolite profiles (S6 Table).

#### Baseline characterization of the two metabolic clusters

At baseline, clusters showed similar anthropometric/clinical parameters and were equally gender distributed (Table 2). Alanine, histidine, lysoPC a C16:0, C18:1, C20:3, C20:4 and C24:0, PC aa C28:1 and PC ae C30:0 were higher in cluster 2 (S7 Table).

**Table 2:** Subjects’ demographic, anthropometric, biochemical and clinical characteristics before bariatric surgery^a^.

	Cluster 1 (n = 24)	Cluster 2 (n = 15)	*P*
Type of surgery, RYGB (nSG)	13 (11)	13 (2)	0.05*
Gender, nF(nM) for RYGB, SG	10 (3), 6 (5)	10 (3), 1(1)	0.73*
Age, y	40.88 ± 9.51	40.67 ± 10.79	0.97
Fasting glucose, mmol/l	107.83 ± 38.05	104.33 ± 19.32	0.80
Fasting insulin, μU/ml	17.17 ± 9.66	18.29 ± 9.14	0.80
HOMA-IR, AU	4.86 ± 3.55	4.94 ± 3.12	0.97
HBA1c, %	5.58 ± 0.21	5.63 ± 0.45	0.84
HBA1c, mmol/mol	37.43 ± 2.30	38.00 ± 4.88	0.84
Weight, kg	136.88 ± 29.82	145.07 ± 22.80	0.42
BMI, kg/m^2^	49.45 ± 9.11	52.23 ± 6.99	0.36
Waist-hip ratio	0.96 ± 0.20	0.89 ± 0.14	0.31
Waist circumference, cm	134.67 ± 21.57	137.29 ± 20.93	0.80
Hip circumference, cm	142.19 ± 21.50	154.43 ± 13.76	0.13
Diastolic pressure, mmHg	83 ± 15	83 ± 12	0.90
Systolic pressure, mmHg	135 ± 20	131 ± 11	0.71
LDL, mg/dl	122.46 ± 34.75	138.13 ± 38.11	0.29
HDL, mg/dl	52.42 ± 20.46	45.79 ± 13.77	0.31
VLDL, mg/dl	25.83 ± 12.48	25.77 ± 10.85	0.99
TG, mg/dl	129.17 ± 62.39	128.87 ± 54.23	0.99
CHOL, mg/dl	202.96 ± 43.95	212.53 ± 44.03	0.60
CRP, mg/l	10.50 ± 8.33	9.66 ± 8.90	0.86
Leptin, mg/ml	78.36 ± 35.73	78.29 ± 36.50	1.00
Adiponectin, ng/ml	7.48 ± 3.75	7.95 ± 4.61	0.84
GOT, U/l	26.75 ± 17.65	19.33 ± 7.04	0.16
GPT, U/l	44.83 ± 28.35	44.07 ± 13.62	0.95
GGT, U/l	32.55 ± 17.78	29.73 ± 17.96	0.74
Uric acid, mmol/l	5.45 ± 1.00	5.59 ± 1.66	0.84
Creatinine, mmol/l	0.80 ± 0.17	0.78 ± 0.11	0.75
Urea, mg/dl	31.29 ± 9.60	31.53 ± 10.43	0.97
Medication use n (%)			
Lipid-lowering drugs	1 (4.16)	0 (0%)	1.00
Antihypertensive drugs	7 (29.16)	2 (13.33)	0.27
Antiinflamatory drugs	3 (12.50)	2 (13.33)	1.00

^a^Values are shown as Mean ± SD otherwise it is indicate. *P* values were determined by independent *t*-test after log-transformated variables

* *P* values were determined by fisher’s exact test

Abbreviations: AU, arbitrary units; BMI, body mass index; CHOL, total cholesterol; CRP, c-reactive protein; GGT, gamma glutamyl transferase; GOT, aspartate transaminase; GPT, alanine transaminase; HbA1c, glycated haemoglobin A1c; HDL, high-density lipoproteins; HOMA-IR, insulin resitance calculated by homeostatic model assessment; LDL, low-density lipoproteins; RYGP, roux-en-Y gastric bypass; SG, sleeve gastrectomy; TG, triglycerides; VLDL, very low-density lipoprotein.

#### Anthropometric/clinical changes over time

Global changes after the surgery in anthropometric/clinical parameters were also reported, independently of the clusters (Table 3). The two clusters were matched by the magnitude of weight loss (~35%) at 6 months versus baseline, but cluster 2 showed a greater decrease in the levels of fasting insulin, HOMA-IR, CHOL, LDL and uric acid, while cluster 1 showed a greater decrease of urea levels (Table 3).

**Table 3:** Anthropometric and clinical characteristics before bariatric surgery and 1, 3 and 6 months after surgery^a^.

	Before surgery	After surgery, months			*P*-Time^b^	*P*-Time X Cluster^b^
		1	3	6		
Fasting glucose, μU/mL	106,49 ± 31,89	89.17 ± 11.25‡	83.76 ± 12.09||	84.31 ± 11.06§	<0.001	0.44
Cl-1	107,83 ± 38,05	90.73 ± 8.03	83.00 ± 13.19	86.00 ± 11.09		
Cl-2	104,33 ± 19,32	86.71 ± 15.03	85.07 ± 10.27	81.85 ± 10.98		
Fasting insulin, μU/mL	17,60 ± 9,36	13.62 ± 8.25	9.94 ± 3.82§	8.95 ± 3.78§	<0.001	<0.05
Cl-1	17,17 ± 9,66	15.71 ± 9.35	10.57 ± 4.23	10.03 ± 3.92		
Cl-2	18,29 ± 9,14	10.33 ± 4.80#	8.83 ± 2.75	7.45 ± 3.12¶		
HOMA-IR, AU	4,89 ± 3,35	3.06 ± 1.99	2.05 ± 0.86||	1.89 ± 0.86||	<0.001	<0.05
Cl-1	4,86 ± 3,55	3.56 ± 2.21	2.15 ± 0.96	2.14 ± 0.87		
Cl-2	4,94 ± 3,12	2.28 ± 1.32¶	1.87 ± 0.65	1.55 ± 0.76		
HBA1c, %	5,59 ± 0,29	5.15 ± 0.26§	5.21 ± 0.29||	5.20 ± 0.23§	<0.001	0.20
Cl-1	5,58 ± 0,21	5.27 ± 0.24	5.23 ± 0.23	5.16 ± 0.17		
Cl-2	5,63 ± 0,45	5.00 ± 0.20	5.19 ± 0.38	5.23 ± 0.28		
HBA1c, mmol/mol	37,60 ± 3,16	32.73 ± 2.81§	33.46 ± 3.22||	33.32 ± 2.51§	<0.001	0.18
Cl-1	37,43 ± 2,30	34.10 ± 2.66	33.68 ± 2.56	32.88 ± 1.83		
Cl-2	38,00 ± 4,88	31.13 ± 2.19	33.16 ± 4.11	33.63 ± 3.01		
Weight, kg	140,03 ± 27,31	124.50 ± 23.49||	113.99 ± 22.31||	100.41 ± 19.53||	<0.001	0.90
Cl-1	136,88 ± 29,82	123.49 ± 26.21	112.68 ± 24.25	99.59 ± 23.25		
Cl-2	145,07 ± 22,80	126.16 ± 19.00	116.25 ± 19.16	101.61 ± 13.11		
BMI, kg/m2	50,52 ± 8,37	45.09 ± 7.19||	41.28 ± 6.80||	36.42 ± 6.14||	<0.001	0.87
Cl-1	49,45 ± 9,11	44.45 ± 7.55	40.55 ± 6.78	36.23 ± 7.09		
Cl-2	52,23 ± 6,99	46.14 ± 6.69	42.53 ± 6.91	36.69 ± 4.66		
Waist-hip ratio, ratio	0,93 ± 0,18	0.87 ± 0.11	0.90 ± 0.21	0.85 ± 0.07	0.27	0.07
Cl-1	0,96 ± 0,20	0.89 ± 0.11	0.86 ± 0.08	0.86 ± 0.07		
Cl-2	0,89 ± 0,14	0.85 ± 0.11	0.97 ± 0.33¶	0.85 ± 0.07		
Waist circumference, cm	135,71 ± 21,05	123.92 ± 16.06||	116.54 ± 13.94||	108.71 ± 13.87||	<0.001	0.80
Cl-1	134,67 ± 21,57	124.30 ± 18.43	116.00 ± 15.33	107.65 ± 15.18		
Cl-2	137,29 ± 20,93	123.29 ± 11.80	117.54 ± 11.42	110.35 ± 11.98		
Hip circumference, cm	147,09 ± 19,53	141.65 ± 14.82	131.84 ± 18.98	125.58 ± 13.60‡	<0.001	0.07
Cl-1	142,19 ± 21,50	140.13 ± 16.43	133.25 ± 15.48	125.45 ± 16.13		
Cl-2	154,43 ± 13,76	144.14 ± 11.84	129.23 ± 24.70¶	125.77 ± 9.02		
Diastolic pressure, mmHg	83,58 ± 14,08	76.47 ± 13.08	78.31 ± 12.46	76.71 ± 9.75	<0.05	0.38
Cl-1	83,95 ± 15,16	76.38 ± 12.46	81.57 ± 11.74	78.05 ± 8.65		
Cl-2	83,00 ± 12,79	76.62 ± 14.55	73.43 ± 12.30	74.58 ± 11.35		
Systolic pressure, mmHg	133,55 ± 17,31	124.41 ± 18.85‡	127.46 ± 16.83	126.26 ± 16.54	0.11	0.19
Cl-1	134,68 ± 20,44	120.86 ± 21.06	129.29 ± 20.13	126.63 ± 19.70		
Cl-2	131,75 ± 11,30	130.15 ± 13.42	124.71 ± 10.19	125.67 ± 10.53		
TG, mg/dL	129,05 ± 58,65	121.89 ± 38.22	109.82 ± 38.63	95.56 ± 38.43	<0.01	0.58
Cl-1	129,17 ± 62,39	127.68 ± 43.08	115.04 ± 42.83	102.00 ± 45.13		
Cl-2	128,87 ± 54,23	112.79 ± 28.06	100.86 ± 29.44	86.15 ± 24.47		
CHOL, mg/dL	206,64 ± 43,66	163.75 ± 36.92||	170.08 ± 37.09§	169.72 ± 45.16‡	<0.001	<0.05
Cl-1	202,96 ± 43,95	170.14 ± 35.42	177.88 ± 35.38	180.58 ± 46.90		
Cl-2	212,53 ± 44,03	153.71 ± 38.29¶	156.71 ± 37.36	153.85 ± 38.85¶		
LDL, mg/dL	128,23 ± 36,32	101.50 ± 29.79	106.35 ± 29.90	106.07 ± 36.70	<0.001	<0.05
Cl-1	122,46 ± 34,75	107.03 ± 27.61	112.66 ± 29.14	113.68 ± 37.35		
Cl-2	138,13 ± 38,11	92.98 ± 32.10¶	95.97 ± 29.18¶	94.65 ± 34.01¶		
HDL, mg/dL	49,97 ± 18,36	38.34 ± 10.16||	44.11 ± 10.89	46.44 ± 13.26	<0.001	0.63
Cl-1	52,42 ± 20,46	40.05 ± 11.88	44.71 ± 11.83	47.89 ± 15.17		
Cl-2	45,79 ± 13,77	35.79 ± 6.41	43.07 ± 9.39	44.31 ± 10.04		
VLDL, mg/dL	25,81 ± 11,73	23.90 ± 7.77	20.50 ± 8.05	18.88 ± 8.30	<0.01	0.44
Cl-1	25,83 ± 12,48	25.74 ± 8.72	22.11 ± 9.24	21.18 ± 9.81		
Cl-2	25,77 ± 10,85	20.68 ± 4.57	17.64 ± 4.49	15.80 ± 4.60		
PCR, mg/l	10,13 ± 8,41	6.69 ± 5.05	5.55 ± 5.46‡	2.55 ± 2.79||	<0.001	0.81
Cl-1	10,50 ± 8,33	6.86 ± 4.70	4.78 ± 2.99	2.42 ± 1.93		
Cl-2	9,66 ± 8,90	6.50 ± 5.70	6.57 ± 7.76	2.69 ± 3.71		
Leptin, mcg/ml	78,33 ± 35,38	41.15 ± 26.40||	32.84 ± 16.28||	23.83 ± 10.28||	<0.001	0.80
Cl-1	78,36 ± 35,73	45.07 ± 33.81	34.21 ± 18.73	23.40 ± 12.67		
Cl-2	78,29 ± 36,50	36.44 ± 13.58	31.16 ± 13.61	24.36 ± 7.04		
Adiponeptin, mcg/ml	7,70 ± 4,11	9.66 ± 4.16	10.98 ± 7.16	12.76 ± 7.71§	<0.001	0.88
Cl-1	7,48 ± 3,75	9.15 ± 3.46	9.94 ± 4.64	12.15 ± 5.42		
Cl-2	7,95 ± 4,61	10.34 ± 5.08	12.41 ± 9.85	13.68 ± 10.66		
GOT, IU/L	23,90 ± 14,84	34.23 ± 17.18‡	27.61 ± 18.79	21.47 ± 9.32	<0.001	0.27
Cl-1	26,75 ± 17,65	36.33 ± 20.05	27.29 ± 22.19	22.11 ± 10.85		
Cl-2	19,33 ± 7,04	31.07 ± 11.67	28.14 ± 11.51	20.54 ± 6.80		
GPT, U/L	44,54 ± 23,56	61.60 ± 28.91§	45.95 ± 25.98	36.22 ± 13.44	<0.001	0.87
Cl-1	44,83 ± 28,35	60.24 ± 25.87	44.21 ± 28.52	35.79 ± 14.73		
Cl-2	44,07 ± 13,62	63.64 ± 33.89	48.93 ± 21.60	36.85 ± 11.86		
GGT, U/L	31,41 ± 17,66	32.31 ± 15.44	30.89 ± 55.01	19.03 ± 9.89‡	<0.001	0.45
Cl-1	32,55 ± 17,78	36.19 ± 12.87	37.67 ± 67.94	21.28 ± 7.50		
Cl-2	29,73 ± 17,96	26.50 ± 17.54	19.29 ± 14.77	15.92 ± 12.12		
Uric, mmol/L	5,50 ± 1,25	6.53 ± 2.52	5.02 ± 1.23	4.46 ± 1.16	<0.001	<0.01
Cl-1	5,45 ± 1,00	6.50 ± 2.94	5.30 ± 1.23	4.82 ± 1.09		
Cl-2	5,59 ± 1,66	6.57 ± 1.75	4.53 ± 1.10	3.95 ± 1.08¶		
Creatinine, mmol/L	0,79 ± 0,15	0.77 ± 0.20	0.67 ± 0.17||	0.72 ± 0.15	<0.001	0.15
Cl-1	0,80 ± 0,17	0.78 ± 0.23	0.65 ± 0.20	0.75 ± 0.17		
Cl-2	0,78 ± 0,11	0.75 ± 0.16	0.69 ± 0.12	0.68 ± 0.11		
Urea, mg/dL	31,38 ± 9,79	21.97 ± 8.09§	23.61 ± 8.28§	25.17 ± 7.96‡	<0.001	<0.05
Cl-1	31,29 ± 9,60	23.09 ± 6.05	22.24 ± 8.23	24.35 ± 8.93		
Cl-2	31,53 ± 10,43	20.21 ± 10.56	25.86 ± 8.13	26.33 ± 6.56		

^a^ Values are shown as Mean ± SD. Total n 39 patients, separate in Cluster 1 (Cl-1, n = 24) and Cluster 2 (Cl-2, n = 15) according K-means clustering at increment 6 month vs baseline. Numbers of serum samples 1, 3 and 6 months after surgery were as follows: Cl-1: 23, 21, 16 and Cl-2 15, 13, 11.

^b^ *P* values represent changes over time (*P*-time) and time x group interaction (*P* -time x group) derived from linear mixed model after log-transformed variables and corrected for multiple testing by the false discovery rate. Taking into account the co-founders: age, gender drug intake and type of surgery.

^‡^ represents p<0.05,

^§^ represents p<0.01 and,

^||^ represents p<0.000 at 1 month, 3 months or 6 months after surgery vs baseline.

^¶^ p<0.05,

^#^ p<0.01 and,

**p<0.0001 represents change over time differently between Cl-1 and Cl-2.

AU, arbitrary units; BMI, body mass index; CHOL, total cholesterol; CRP, c-reactive protein; GGT, gamma glutamyl transferase; GOT, aspartate transaminase; GPT, alanine transaminase; HbA1c, glycated haemoglobin A1c; HDL, high-density lipoproteins; HOMA-IR, insulin resitance calculated by homeostatic model assessment; LDL, low-density lipoproteins; TG, triglycerides; VLDL, very low-density lipoprotein.

#### Correlations between metabolic changes and anthropometric/clinical changes

To identify potential links between anthropometric/clinical and metabolic variables, and to elucidate metabolic differences between clusters, the next step was to correlate anthropometric/clinical variables with metabolites in T6–T0 for each cluster.

Fig 2B presents the correlation of cluster 1 between changes in clinical variables and discriminant metabolites of the clusters (Table 1). Those metabolites that were not the most discriminant between clusters but also correlated were also included in the network. Cluster 2 did not present statistical significance in these metabolites. S3A and S3B Fig correlate discriminant metabolites in cluster 1 and cluster 2 respectively.

The most metabolite classes in each metabotype are shown in S4A and S4B Fig. Both phenotypes present major changes in unsaturated PC ae and PC aa and SMs. The impact of changes in aromatic amino acids and BCAAs were also significant in cluster 1 whereas in cluster 2, saturated and unsaturated lysoPC and the amino acids and BCAAs did not reach statistical significance. Most of the metabolites that compose these groups of compounds decreased in cluster 2, but increased in cluster 1, except aromatic amino acids and the BCAAs, that decreased in both clusters. Data are shown in S8 Table.

## Discussion

No clinical or quantified metabolite confirmed the assumption that the “metabolically unhealthy” and “metabolically healthy” phenotypes respond differently over time to bariatric surgery. Therefore, we believe that the impact of bariatric surgery on the metabolic status is so intense that any initial metabolic differences are nullified.

Our results highlight shared and exclusive profiles of the response to surgery among the subjects throughout 6 months of follow-up, regardless of the baseline metabolic state, gender and age of the subjects and the surgical procedure received.

Overall, the morbidly obese traits almost vanished 6 months after surgery and the metabolic status of the subjects improved, thereby confirming previous reports [19]. For the first time, changes in metabolites at the increment T6–T0 indicated two potentially different metabolic phenotypes (metabotypes) of short-term adaptation to bariatric surgery, hereinafter metabotype 1 (cluster 1) and metabotype 2 (cluster 2). Although clusters were clinically similar at baseline, remarkable differences in the post-surgery progression were observed. Metabotype 2 presented a greater degree of improvement in fasting insulin, HOMA-IR index, total cholesterol, LDL and uric acid 6 months after surgery, whereas metabotype 1 showed lower levels of urea, suggesting over- and down-expression of specific metabolic pathways in each phenotype.

### Amino acid remodelling after bariatric surgery

#### Shared trends after surgery between metabotypes

In line with other works, an increase of branched-chain amino acids (BCAAs, isoleucine, leucine and valine) and aromatic amino acids has been described in obesity and they have been proposed as biomarkers for screening the risk of developing diabetes [20]. This study reports that bariatric surgery remodels amino acid metabolism as early as in the first month post-surgery. An increase in the catabolic enzymes of the BCAAs metabolism in subcutaneous and visceral fat depots after bypass procedure could explain this phenomenon [21]. Recently, it has found that after the intervention, microbial functions involved in pathways of production of these amino acids are more similar to lean subjects [22].

Our study also mirrored changes in urea-nitric oxide (NO) metabolism, specially by changes in ornithine, arginine and symmetric dimethylarginine levels. A recent study pointed a relationship between arginine and its methylated products (asymmetric and symmetric dimethylarginine) and adverse cardiovascular events and all-cause mortality [23]. We also found that these metabolites change differently according to the phenotype of the subject.

In accordance with others [24], we observed an increase in the glycine levels after surgery. Low levels of glycine have been detected in patients with a high risk of developing diabetes, caused by an increase in glutathione consumption [25], therefore this may be an indicator of glycaemic control.

#### Distinctive hallmarks after surgery between metabotypes

Our results show an important association between the increase of insulin sensitivity and the decrease of uric acid levels. Hyperuricemia has been described as a causal factor of insulin resistance due to gluconeogenesis disruption [26]. Paradoxically, the identification of individuals with hyperuricemia and a balanced glycaemia homeostasis indicates a pluri-mechanistic connection between both conditions [27].

Uric acid stimulates hepatic gluconeogenesis [28]. After surgery, uric acid led to the downregulation of the gluconeogenesis in metabotype 1, mirrored by the restoration of the amino acids metabolism via the glucose-alanine cycle, also observed by others [29]. Metabotype 2 had lower levels of alanine and glycogenic precursors. Hence, metabotype 2 may fail to compensate for the peripheral glucose demand after surgery, resulting in a greater hypoglycaemic state, also indicated in [30]. Previous studies prove that the reduction of glycogenic substrates is independent of the magnitude of weight loss [31].

Metabotype 1 presented higher levels of compounds of the glucose-alanine and urea cycles at 6 months post-surgery. However, the low levels of urea and the negative correlation between arginine and uric acid in metabotype 1 suggest that bariatric surgery inhibited the production of urea-NO and arginine accumulation [32]. Jia et al. showed that beta-cells treated with uric acid triggered an inflammatory response and the overproduction of NO [33]. We observed that after the surgery this would reverse, uric acid decreased and methylated products of arginine metabolism increased after bariatric surgery in metabotype 1.

These observations also emphasize that bariatric surgery engages multiple mechanisms, independent and dependent of NO [23].

### Lipid metabolism remodelling after bariatric surgery

For the first time, changes in the PCs profile have been related to the improvement of insulin resistance and uricemia after bariatric surgery, independently of the degree of weight loss. In fact, a recent study has described the protective role of phospholipids in pancreatic islets [34]. However, the interplay between PCs and uric acid after bariatric surgery and a possible connection with insulin resistance has not been reported yet.

Although previous reports did not identify an association between uric acid and PCs, experimentally have been demonstrated that uric acid produces oxidative stress in mitochondria, inhibits the aconitase enzyme in the tricarboxylic acid cycle and impairs the beta-oxidation of fatty acids [35].

The high levels of acetyl-CoA reflect this low capacity of the tricarboxylic acid cycle for beta-oxidation of long-chain fatty acids and an increase in carbohydrate catabolism [36]. The carnitine metabolism disruption is within the crosstalk between the alteration of the glucose and lipid metabolism. It is worth noting that the levels of carnitines C2, C14:1 and C18:1 immediately increased 1 month post-surgery. Thus they mirror acute changes in the metabolism after the surgery rather than carnitine synthesis or dietary origin, as fasting serum was used to reduce the dietary effect, and the levels of lysine and methionine—precursors of carnitine synthesis—remained stable after the surgery.

Large-scale metabolomics studies have demonstrated the association of obesity, high insulin resistance and dyslipidaemia with specific lipids such as SMs and diacyl-PCs [37]. However, few studies have dissected their individual contribution to adipose tissue expansion and/or insulin resistance. Our findings demonstrate beneficial effects of bariatric surgery on metabolic health by the restoration of the sphingolipid-phospholipid metabolism, through the improvement of the lipoprotein profile. (Lyso)PCs have been considered a pathognomonic characteristic of subjects with obesity and high insulin resistance [38]. LysoPCs are produced by the hydrolysis of PCs during the oxidation of LDL [39] or by the action of lecithin-cholesterol acyltransferase. Therefore, the decreased levels of lysoPCs after surgery observed in metabotype 2 could be explained by a reduction of these biochemical processes [40]. Controversially, other authors stated that there was a decrease in lysoPC levels in insulin resistance subjects with non-alcoholic fatty liver [41], reflecting the role of lysoPCs as lipid-signalling molecules in the homeostasis of glucose [42]. Contrary to expected, PCs and lysoPCs presented similar trends in both phenotypes, suggesting that other mechanisms rather than the over-/downregulation of lysoPCs production from PCs could be modified after surgery.

Oxidized SMs enhance the susceptibility of HDL to aggregation and accumulation in the arterial walls [43] and reduce their clearance. A recent study reported that weight loss after a hypocaloric-diet intervention was associated with a decrease of HDL-SM, together with an improvement of the metabolic status [43]. The different post-surgery metabolic profiles of phenotypes 1 and 2 suggest that the benefits from the bariatric surgery are phenotypically dependent.

Taking all these results together, this study reveals that individuals that experienced a greater improvement of insulin resistance and cardiovascular factors after bariatric surgery had lower levels of gluconeogenic precursors, metabolites of the urea metabolism associated with NO mechanisms, and changes in the microcomponents of lipoproteins.

This short longitudinal design of the study allowed us to explore the earliest changes after surgery, but not changes due to adaptation. Although the MFA showed different post-surgery trends, the low number of patients in the study limited the amount of clusters and members in each one. The groups, formed by K-means cluster analysis,were unbalanced in terms of type of surgery. K-means is an unsupervised method and we could not address this point in the analysis. Consequently, despite considering type of surgery as a co-founder, we cannot affirm that the differences observed along the time are independent of the type of surgery. Several statistical approaches corroborated the results, notwithstanding, an independent cohort and prospective study is needed to confirm them. This work presents an innovative metabolomics and statistical approach to study the metabolic adaptations of patients after bariatric surgery. We have expanded the quantification of the metabolites-metabolic pathways, providing a general perspective and a dissection of the multiple responses to bariatric surgery.

## Conclusions

Our data allowed the identification of metabotypes that are more likely to benefit from bariatric surgery, independently of the baseline status of the patients. The complexity of the metabolome requires complementary analytical methodologies in order to expand its coverage for a better understanding of the biological processes. Moreover, other factors such as global lifestyle or even a genetic component might be able to explain these metabotypes. Finally, to go beyond the analysis of individual mechanisms to the study of systems biology from an integral view, further investigation is needed.

## Supporting information

S1 File Multiple factor analysis and cluster analysis. (DOC)

S1 Fig Multifactor analysis graphical outputs. A. Eigenvalues of the different principal components (PCx) of the analysis. B. Plot for groups of variables versus the two first principal components. C. Partial axes of each group in the first two PCx. The increment 1 month—baseline is highly correlated with the first dimension and negatively correlated with the second dimension. The increment 3 months—1 month is negatively correlated with the first dimension. The increment 6 months—3 months is positively explained by the first component and second component.
(TIF)

S2 Fig Scatter plot of the metabolically healthy obese (red dots) and metabolically unhealthy (blue triangles) individuals in the following increments: A: T1–T0, B: T3–T0 and C: T6–T0. (TIF)

S3 Fig Metabolic correlation matrix of the discriminant metabolites of the K-means cluster analysis at the increment T6-T0 A) correlations between metabolites in phenotype 1.B) correlations between metabolites in phenotype 2. Only those correlations with p<0.05 are shown.
(TIF)

S4 Fig Bubble plot of the most impacted metabolite clusters in metabotype 1 (A) and 2 (B) respectivetly. Chemical enrichment statistics was calculated by applying the Kolmogorov-Smirnov test on the metabolites at the increment of time T6-T0. Clusters are generated by chemical similarity and ontology mapping. Cluster colors give the proportion of increased or decreased compounds (red = increased, blue = decreased). *P* values were corrected for multiple testing by false discovery rate and only those clusters with p<0.05 are shown.
(TIF)

S1 Table Summary of the metabolites (semi-)quantified in blood serum by MS/MS and those excluded. **Metabolites are grouped into classes based on their metabolic function or structural similarities**. According to manufacturer guidelines the detected MRM signal for lipid measurements is a sum of several isobaric/isomeric lipids. "For example: the signal of PC aa C36:6 can arise from at least 15 different lipid species that have different fatty acid composition (e.g. PC 16:1/20:5 versus PC 18:4/18:2), various positioning of fatty acids sn-1/sn-2 (e.g. PC 18:4/18:2 versus PC 18:2/18:4) and different double bond positions and stereochemistry in those fatty acid chains (e.g. PC(18:4(6Z,9Z,12Z,15Z)/18:2(9Z,12Z)) versus PC (18:4(9E,11E,13E,15E)/18:2(9Z,12Z)))". QC-CV>25%: high analytical variances in the quality control replicates (coeficient of variation >25%); LOD: limit of detection; LLOQ: limit of quantification.
(XLS)

S2 Table Anthropometric, biochemical and clinical characteristics of metabolically healthy (MH) and unhealthy (MU) individuals before the intervention^1^. ^1^ Values are shown as Mean ± SD. *P* values were determined by independent t-test after log-transformated the variables * *P* values were determined by fisher’s exact test. AU, arbitrary units; BMI, body mass index; BP, blood pressure; CHOL, total cholesterol; C- LDL, low-density lipoproteins cholesterol; C-HDL, high-density lipoproteins cholesterol; CRP, C-reactive protein; DBP, diastolic blood pressure; GOT, aspartate transaminase; GPT, alanine transaminase; GGT, gamma glutamyl transferase; HbA1c, glycated haemoglobin A1c; HOMA-IR, insulin resitance calculated by homeostatic model assessment;; RYGP, Roux-en-Y gastric bypass SBP, systolic blood pressure; SG, sleeve gastrectomyTG, triglycerides. Cardiometabolic risk factors Adult Treatment Panel III criteria): Waist circumference >102 cm for male and >88 for female; TG over 150 mg/dl; HDL ≤40 for male and ≤50 for female; BP, SBP>130mmHg or DBP>85mmHg; fasting glucose over 110 mmol/ml.
(XLS)

S3 Table Concentrations of metabolites in metabolically healthy (MH) and unhealthy (MU) individuals before the intervention^1^. ^1^Values are shown as Mean ± SD (μM) ^2^ *P* values derived from t-test after log-transformed the variables, p-adjusted are *P* values corrected for multiple testing by the false discovery rate aa, acyl-acyl; ae, acyl-alkyl; LPC a, lysophosphatidylcholines; Cx:y, where x is the number of carbons in the fatty acid side chain; y is the number of double bonds in the fatty acid side chain; DC, decarboxyl; M, methyl; OH, hydroxyl; PC, phophatidylcholine; SM, sphingomyelin.
(XLS)

S4 Table Anthropometric, biochemical and clinical characteristics of metabolically healthy (MH) and unhealthy (MU) individuals over time^1^. ^1^Values are shown as Mean ± SD. Total n 39 patients, separate in metabollicaly health (MH, n = 21) and metabollicaly abnormal (MU, n = 18). ^2^*P* values represent changes over time (p-time) and time x group interaction (p-time x group) derived from linear mixed model after log-transformate the variables and corrected for multiple testing by false discovery rate. Taking into acount the co-founders: age, gender, drug intake and type of surgery. *, **,*** represents p<0.05, p<0.01 and p<0.0001 respectivetly at 1 month, 3 months or 6 months after surgery vs baseline estimated in linear mixed-effects models, corrected for multiple testing by the false discovery rate. #, p<0.05; ##, p<0.01 and ###, p<0.0001 represents change over time differently between MH and MU. AU, arbitrary units; BMI, body mass index; CHOL, total cholesterol; C- LDL, low-density lipoproteins cholesterol; C-HDL, high-density lipoproteins cholesterol; CRP, c-reactive protein; GOT, Aspartate transaminase; GPT, Alanine transaminase; GGT, Gamma glutamyl transferase; HbA1c, glycated haemoglobin A1c; HOMA-IR, insulin resitance calculated by homeostatic model assessment; TG, triglycerides.
(XLS)

S5 Table Concentration of metabolites of metabolically healthy (MH) and unhealthy (MU) individuals after surgery^1^. ^1^Values are shown as Mean ± SD. Total n 39 patients, separate in metabollicaly health (MH, n = 21) and metabollicaly abnormal (MU, n = 18).^2^ *P* values represent changes over time (p-time) and time x group interaction (p-time x group) derived from linear mixed model after log-transformed variables and corrected for multiple testing by false discovery rate. Taking into account the co-founders: age, gender, drug intake and type of surgery. #, p<0.05; ##, p<0.01 and ###, p<0.0001 represents change over time differently between MH and MU. aa, acyl-acyl; ae, acyl-alkyl; LPC a, lysophosphatidylcholines; Cx:y, where x is the number of carbons in the fatty acid side chain; y is the number of double bonds in the fatty acid side chain; DC, decarboxyl; M, methyl; OH, hydroxyl; PC, phophatidylcholine; SM, sphingomyelin.
(XLS)

S6 Table Changes in metabolite concentrations of both clusters after surgery. ^1^Values are shown as Mean ± SD. Total n 39 patients, separate in Cluster 1 (Cl-1, n = 24) and Cluster 2 (Cl-2, n = 15) according K-means clustering at increment 6 month vs baseline. ^2^ *P* values represent changes over time (p-time) and time x group interaction (p-time x group) derived from linear mixed model after log-transformed variables and corrected for multiple testing by the false discovery rate. Taking into account the co-founders: age, gender, drug intake and type of surgery. #, p<0.05; ##, p<0.01 and ###, p<0.0001 represents change over time differently between Cl-1 and Cl-2.
(XLS)

S7 Table Concentrations of metabolites in both clusters before the intervention. ^1^Values are shown as Mean ± SD ^2^*P* derived from t-test after log-transformed variables and corrected for multiple testing by false discovery rate.
(XLS)

S8 Table Raw data with clinical and metabolomic variables in each period of time. (CSV)
