# Supplementary material for: Metabotypes of response to bariatric surgery independent of the magnitude of weight loss
Source: PLoS One. 2018 Jun 1;13(6):e0198214. doi: 10.1371/journal.pone.0198214 (PMC5983508; doi:10.1371/journal.pone.0198214)
Supplement: S1 File — (DOC) [file pone.0198214.s001.doc]

**Supporting information**

**Metabotypes of response to bariatric surgery independent of the magnitude of weight loss**

Magali Palau-Rodriguez^1,2^, Sara Tulipani^1,3^,Anna Marco-Ramell^1,2^, Antonio Miñarro^4^, Olga Jáuregui^1,5^, Alex Sanchez-Pla^4,6^, Bruno Ramos-Molina^3,7^, Francisco J Tinahones^3,7^, Cristina Andres-Lacueva^1,2^ *

^1^ Biomarkers & Nutrimetabolomic Laboratory, Nutrition, Department of Food Science and Gastronomy, XaRTA, INSA-UB, Campus Torribera, Faculty of Pharmacy and Food Science, University of Barcelona, Barcelona, Spain

^2^ CIBER Fragilidad y Envejecimiento Saludable [CIBERfes], Instituto de Salud Carlos III [ISCIII], Madrid, Spain

^3^ Biomedical Research Institute [IBIMA], Service of Endocrinology and Nutrition, Malaga Hospital Complex [Virgen de la Victoria], Málaga, Spain

^4^ Department of Genetics, Microbiology and Statistics, Faculty of Biology, University of Barcelona, Barcelona, Spain

^5^ Scientific and Technological Centers of the University of Barcelona (CCIT-UB), Barcelona, Spain

^6^ Statistics and Bioinformatics Unit, Vall d'Hebron Institut de Recerca [VHIR], Barcelona, Spain

^7^ CIBER Fisiopatología de la Obesidad y Nutrición [CIBERobn], Instituto de Salud Carlos III [ISCIII], Barcelona, Spain

Corresponding author:

*Cristina Andres-Lacueva

Biomarkers & Nutrimetabolomic Lab, Nutrition & Food Science Dept, XaRTA, INSA, Campus Torribera, CIBERFES, Pharmacy and Food Science Faculty, University of Barcelona, 08028, Spain.

Phone: (+34) 934034840

Fax: (+34) 934035931

E-mail address: candres@ub.edu

**Supplementary Methods**

***Multiple factor analysis***

Multiple factor analysis or ‘multiple factorial analyses’ (MFA) is an unsupervised multivariate technique, such as principal component analysis (PCA), which aims to separate the data into principal components (PCx). Each PC[[1]](#footnote-2) contributes to explaining the variability of the data[[2]](#footnote-3) with several variables correlated in each one. Therefore, the more information that is described with the fewest number of PCs, the better the variables describe the model. In the same way, MFA and PCA can also be graphically displayed, plotting the projections of the observations and the loadings of the variables onto the components (the so-called ‘circle of correlations’).

However, when the observations are described by several groups with a different number of variables in each one − subsets of data − e.g. on longitudinal studies, to perform a PCA could be erroneous, giving weight to the set of variables with the largest number and not to the one that better explains the model. To solve this problem, MFA is able to combine multiple data sets regardless of the quantity and nature − quantitative/qualitative − of the variables ^1^. In fact, MFA could be employed in several disciplines ^2^ and contexts ^3^.

Two steps are necessary to perform an MFA: First, for each subset of variables a PCA is computed if the variables are quantitative; or a multiple correspondence analysis (MCA) if the variables are qualitative. This will be followed by the normalization of each data table, dividing all its elements by the first singular value obtained from its PCA (the square root of an eigenvalue can be considered a variance) to make variables comparable.

The second step is to build up a grand matrix with all normalized data tables, analysed by a (non-normalized) PCA, which gives a set of factor scores for the observations and loadings of each variable. In addition, MFA provides a set of partial factor scores for the observations of each data table that reflects the specific ‘viewpoint’ of this data table ^4^. New variables are created as linear combinations of the original variables and can be interpreted using variables’ correlations or contributions together with a set of graphical outputs.

Overall, different entities are represented sharing dimensions:

1. Plot for groups of variables versus the two first principal components.
2. Individual factor map represents the observations with the partial values of each group.
3. Variables correlation circle, in which the circle’s radius represents how the variable (radius) is correlated in each PC (axes). The more a vector has a magnitude close to 1, the better the projection is.
4. Partial axes of each group in the first two PCs.

***Clustering analysis***

One common statistical step in biomedicine studies is to group objects by similarity − clustering analysis. Several methods are available to fit different inputs. However, there is not a general best-performing approach, it depends on the data set ^5^.

Most of the algorithms require the user to identify the number of clusters before the clustering analysis. Foreseeably, this step is not straightforward and different procedures could be used to address it. In 1985, Milligand and Cooper evaluated different cluster solutions, suggesting the Calinski-Harabasz index (1974) as one of the most effective ^6^.

The Calinski-Harabasz index ensures the compactness and isolation of the cluster through the minimum sum of distances between the objects of the cluster and their fictitious cluster centre − the centroid − and the maximum distance between the clusters, based on the average between- and within- cluster sum of squares.

Moreover, as an unsupervised learning task, one of the most important issues is to evaluate the goodness of the data partition. This is possible through internal and external clustering validation indexes. Internal validation would judge the cluster quality on the basis of certain intrinsic statistical properties of the clustering itself ^7^, without any external information not present in the data that external validation relies upon ^8^. In the same way, different measures are equivalent to deal with the validation step. In this study, the Calinski-Harabasz index would solve internal validation.

**References**

1. Escofier, B. and Pagès J. Analyses factorielles simples et multiples: objectifs, méthodes, interprétation. 1990.

2. Lalloué B, Monnez J-M, Padilla C, Kihal W, Zmirou-Navier D, Deguen S. Data analysis techniques: a tool for cumulative exposure assessment. *J Expo Sci Environ Epidemiol*. 2014;(February):1-9. doi:10.1038/jes.2014.66.

3. Dumas ME, Canlet C, Debrauwer L, Martin P, Paris A. Selection of biomarkers by a multivariate statistical processing of composite metabonomic data sets using multiple factor analysis. *J Proteome Res*. 2005;4(5):1485-1492. doi:10.1021/pr050056y.

4. Abdi H, Williams LJ, Valentin D. Multiple factor analysis: principal component analysis for multitable and multiblock data sets. *Wiley Interdiscip Rev Comput Stat*. 2013;5(2):149-179. doi:10.1002/wics.1246.

5. Andreopoulos B, An A, Wang X, Schroeder M. A roadmap of clustering algorithms: Finding a match for a biomedical application. *Brief Bioinform*. 2009;10(3):297-314. doi:10.1093/bib/bbn058.

6. Milligan GW, Cooper MC. An examination of procedures for determining the number of clusters in a data set. *Psychometrika*. 1985;50(2):159-179. doi:10.1007/BF02294245.

7. Liu Y, Li Z, Xiong H, Gao X, Wu J. Understanding of intenal clustering validation measures. *IEEE Internatinal Conf Data Min*. 2010:911-916. doi:10.1109/ICDM.2010.35.

8. Wu J, Xiong H, Chen J. Adapting the right measures for K-means clustering. *Proc 15th ACM SIGKDD Int Conf Knowl Discov data Min - KDD ’09*. 2009:877. doi:10.1145/1557019.1557115.

1. These variables are called, depending upon the context, principal components (PCs), factors, eigenvectors, singular vectors or loadings. [↑](#footnote-ref-2)
2. The importance of each component is expressed by the variance (i.e. eigenvalue) of its projections or by the proportion of the variance explained. In this context, PCA is interpreted as an orthogonal decomposition of the variance (also called inertia) of a data table. [↑](#footnote-ref-3)
