# Supplementary figures and images for: Metabotypes of response to bariatric surgery independent of the magnitude of weight loss
Source: PLoS One. 2018 Jun 1;13(6):e0198214. doi: 10.1371/journal.pone.0198214 (PMC5983508; doi:10.1371/journal.pone.0198214)

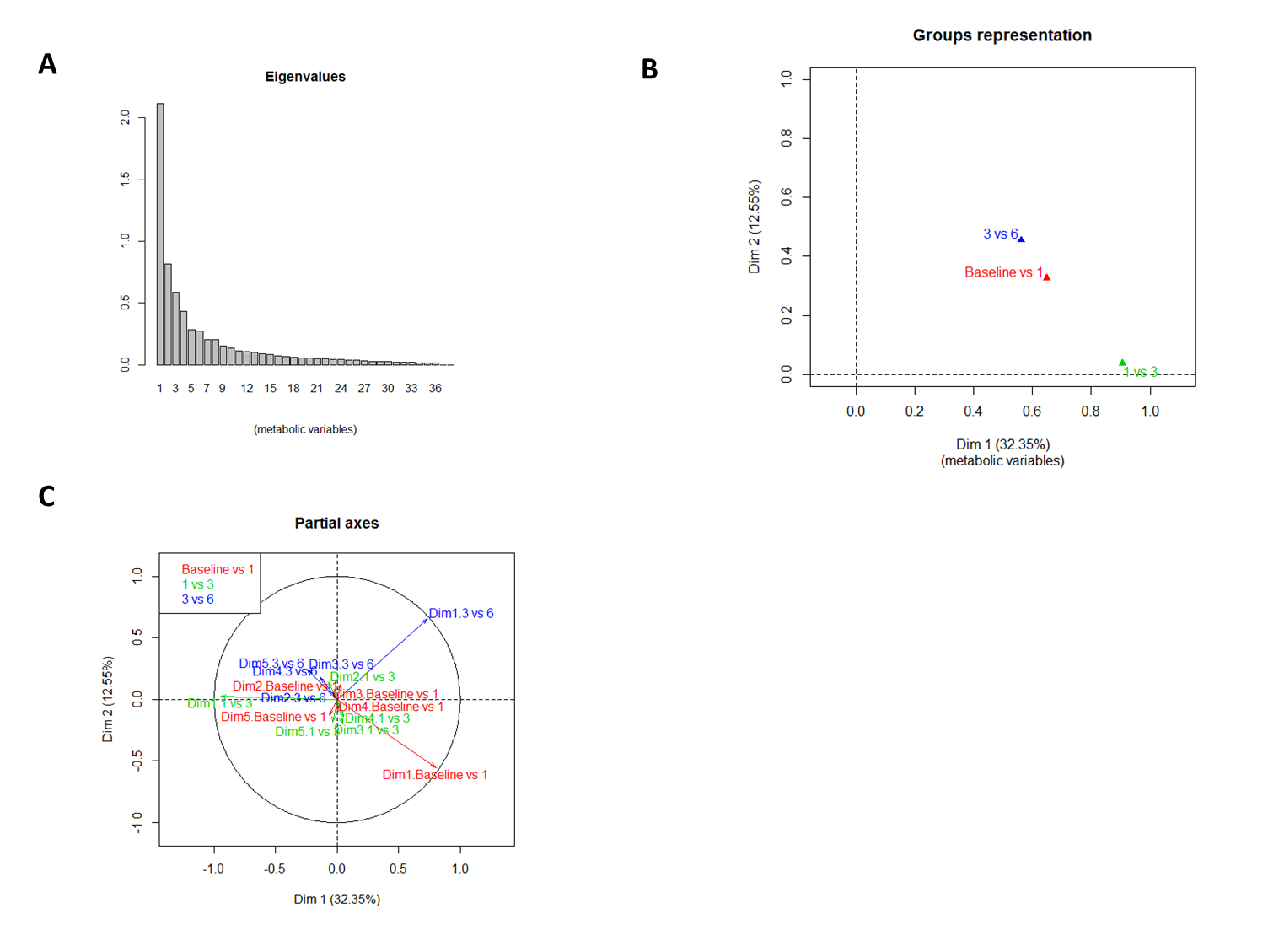

Supplement: S1 Fig — A. Eigenvalues of the different principal components (PCx) of the analysis. B. Plot for groups of variables versus the two first principal components. C. Partial axes of each group in the first two PCx. The increment 1 month—baseline is highly correlated with the first dimension and negatively correlated with the second dimension. The increment 3 months—1 month is negatively correlated with the first dimension. The increment 6 months—3 months is positively explained by the first component and second component. (TIF) [file pone.0198214.s002.tif]

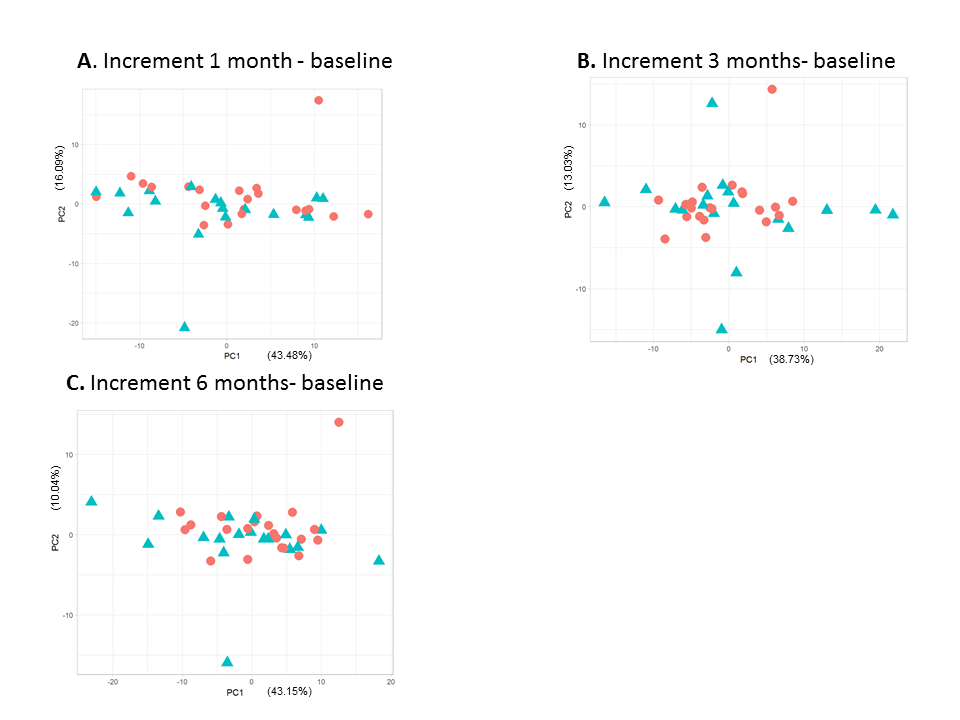

Supplement: S2 Fig — (TIF) [file pone.0198214.s003.tif]

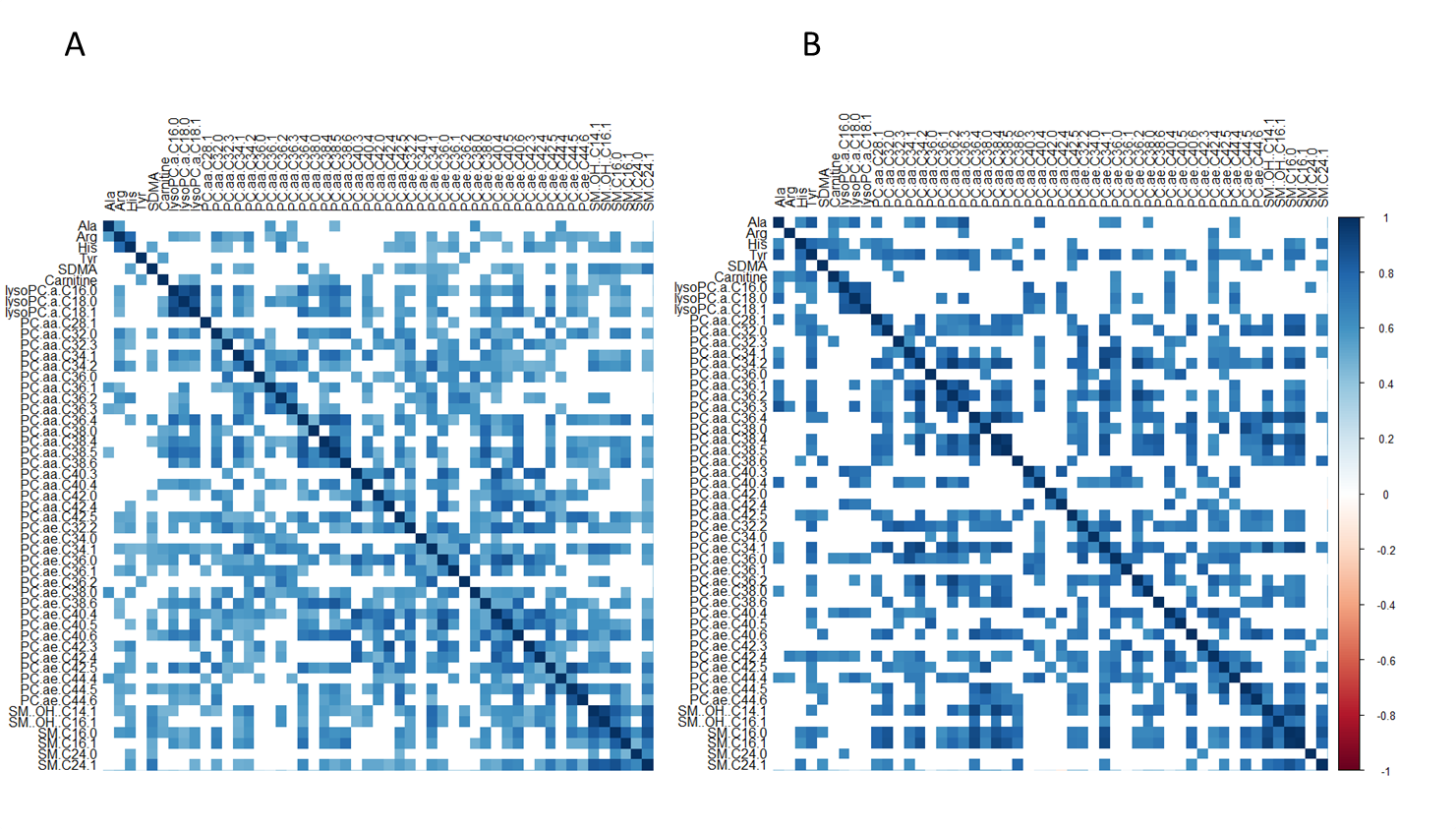

Supplement: S3 Fig — Only those correlations with p<0.05 are shown. (TIF) [file pone.0198214.s004.tif]

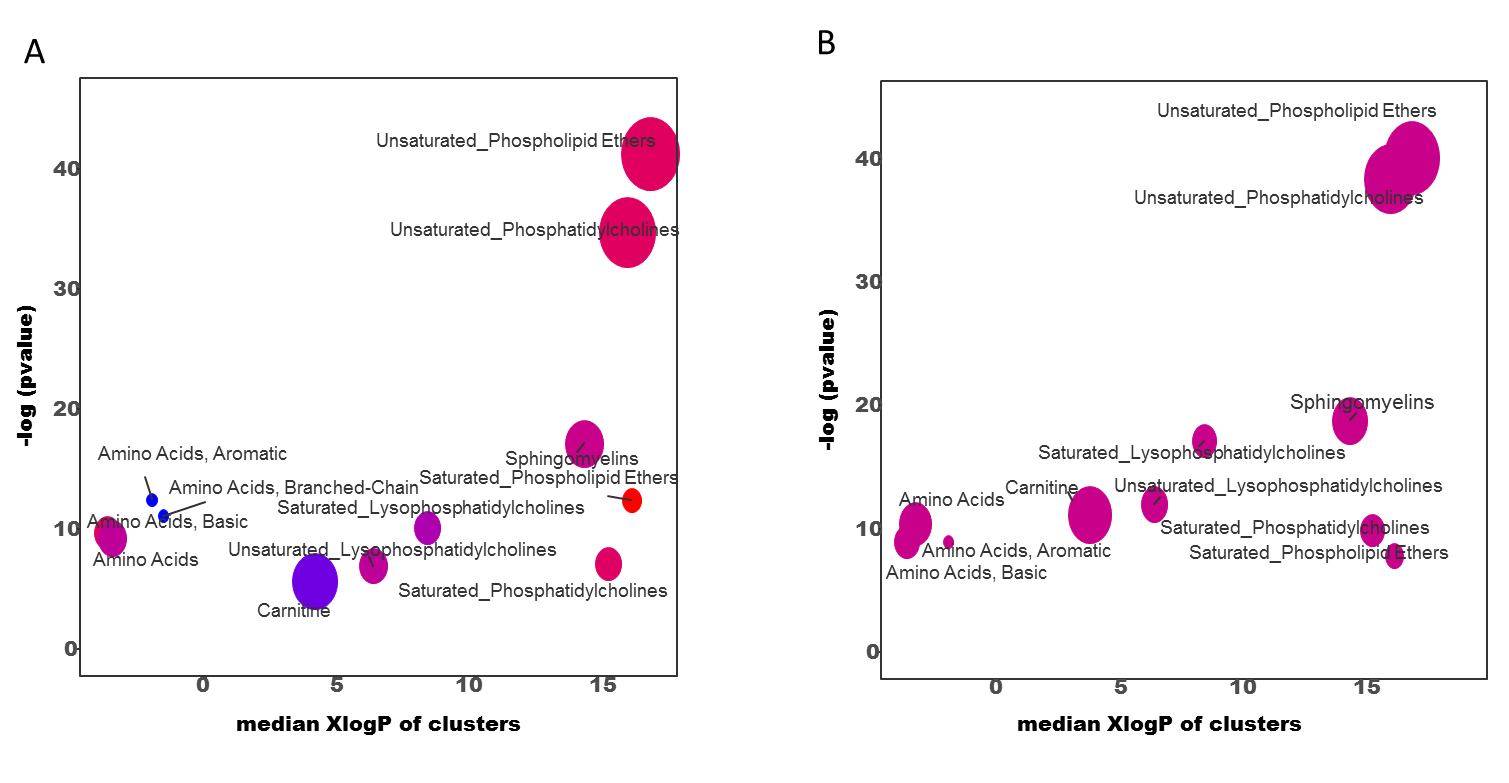

Supplement: S4 Fig — Chemical enrichment statistics was calculated by applying the Kolmogorov-Smirnov test on the metabolites at the increment of time T6-T0. Clusters are generated by chemical similarity and ontology mapping. Cluster colors give the proportion of increased or decreased compounds (red = increased, blue = decreased). P values were corrected for multiple testing by false discovery rate and only those clusters with p<0.05 are shown. (TIF) [file pone.0198214.s005.tif]
